# Dissemination of *Pseudomonas aeruginosa bla*_NDM-1_-Positive ST308 Clone in Singapore

**DOI:** 10.1128/spectrum.04033-22

**Published:** 2023-04-12

**Authors:** Sai Rama Sridatta Prakki, Pei Yun Hon, Ze Qin Lim, Natascha May Thevasagayam, Song Qi Dennis Loy, Partha Pratim De, Kalisvar Marimuthu, Shawn Vasoo, Oon Tek Ng

**Affiliations:** a National Centre for Infectious Diseases, Singapore; b Tan Tock Seng Hospital, Singapore; c Yong Loo Lin School of Medicine, National University of Singapore and National University Health System, Singapore; d Lee Kong Chian School of Medicine, Nanyang Technological University, Singapore; University of Manitoba

**Keywords:** carbapenemase, *Pseudomonas aeruginosa*, ST308, region-specific, whole-genome sequencing, chromosomal integration

## Abstract

Pseudomonas aeruginosa ST308 clone has been reported to carry carbapenemase genes such as *bla*_IMP_ and *bla*_VIM_ but has been rarely associated with *bla*_NDM-1_. A total of 199 P. aeruginosa ST308 clinical and environmental isolates obtained between April 2019 and November 2020 from a tertiary-care hospital in Singapore were characterized using whole-genome sequencing. In addition, 71 *bla*_NDM-1_-positive ST308 whole-genome sequences from two other local tertiary-care hospitals in Singapore and 83 global *bla*_NDM-1_-negative ST308 whole-genome sequences in public databases were included to assess phylogenetic relationships and perform genome analyses. Phylogenetic analysis and divergent time estimation revealed that *bla*_NDM-1_-positive P. aeruginosa ST308 was introduced into Singapore in 2005 (95 % highest posterior density: 2001 to 2008). Core genome, resistome, and analyses of all local *bla*_NDM-1_-positive ST308 isolates showed chromosomal integration of multiple antibiotic resistance genes (ARGs) [*aac(3)-Id*, *aac(6′)-Il*, *aadA6*, *aadA11*, *dfrB5*, *msr*(*E*), *floR*, *sul2*, and *qnrVC1*], which was absent in global *bla*_NDM-1_-negative ST308 sequences. Most ARGs and virulence genes were conserved across isolates originating from the three different local hospitals. Close genetic relatedness of the *bla*_NDM-1_-positive ST308 clinical and environmental isolates suggests cocirculation between the hospital environment and human hosts with the hospital environment as a potential reservoir. Core genome single nucleotide polymorphism analyses revealed possible clonal transmission of *bla*_NDM-1_-positive ST308 isolates between the three hospitals over 7 years. Bloodstream isolates accounted for six of 95 (6.3%) clinical isolates. This study reports the introduction of a pathogenic *bla*_NDM-1_-positive P. aeruginosa ST308 more than a decade ago in Singapore and warrants surveillance for wider dissemination.

**IMPORTANCE** P. aeruginosa is a Gram-negative opportunistic pathogen ubiquitously found in the environment and a major cause of nosocomial infections. While the P. aeruginosa ST308 clone has been known to bear *bla*_IMP_ and *bla*_VIM_ among global isolates, reports of *bla*_NDM-1_-positive P. aeruginosa ST308 are rare. The local *bla*_NDM-1_-positive P. aeruginosa ST308 isolates detected in this study appear to be unique to this region, with evidence of chromosomal acquisition of multiple ARGs compared to global *bla*_NDM-1_-negative P. aeruginosa ST308 isolates. Surveillance in Singapore and beyond for dissemination is essential to determine whether existing measures are sufficient to control the spread of this ST308 clone.

## INTRODUCTION

Pseudomonas aeruginosa, a Gram-negative opportunistic pathogen ubiquitously found in the environment, is a major cause of nosocomial infections (1–3). The population structure of P. aeruginosa has generally been conceptualized as panmictic, with a large number of genotypes that frequently recombine (4). However, recent reports have increasingly shown that nosocomial multidrug resistant (MDR) and extensively drug-resistant (XDR) P. aeruginosa strains are not panmictic but largely characterized by 10 main high-risk clones (sequence type 235 [ST235], ST111, ST233, ST244, ST357, ST308, ST175, ST277, ST654, and ST298).

In Singapore, recent reports from two separate institutions have documented the emergence of *bla*_NDM-1_-positive P. aeruginosa ST308, with the earliest isolate identified in 2013 (5, 6). A prior survey of 2,552 nonduplicate P. aeruginosa isolated in 2008 from a major hospital in Singapore identified 11 metallo-β-lactamase positive isolates, all of which carried either *bla*_IMP_ or *bla*_VIM_ with no *bla*_NDM-1_-positive isolates identified and no predominant ST (7). Globally, ST308 has been known to bear *bla*_IMP_ and *bla*_VIM_, and the only published report of *bla*_NDM-1_-positive P. aeruginosa ST308 to our knowledge was from the neighboring country of Malaysia, where a single isolate was identified (8).

From 2019 to 2020, we detected and whole-genome sequenced *bla*_NDM-1_-positive P. aeruginosa ST308 in our institution from rectal swabs, clinical samples, and environmental samples. Combining our genomic data with the publicly available genome sequences and metadata from two other local hospitals, we aimed to determine the phylodynamics and evolution of *bla*_NDM-1_-positive P. aeruginosa ST308 in Singapore. In addition, we examined the resistome and virulome of *bla*_NDM-1_-positive P. aeruginosa ST308 and the relationship of clinical and environmental isolates.

## RESULTS

### Bacterial isolates.

A total of 199 *bla*_NDM-1_-positive P. aeruginosa isolates were collected from hospital C between April 2019 and November 2020, 183 of which were patient isolates, while 16 were from environmental sampling. Among the patient isolates 88 were from surveillance samples, with urine (*n* = 48) being the most common clinical isolate sample type (see Table S1 in the supplemental material). Six samples were isolated from blood. The median age of the patients was 76 years (interquartile range, 65 to 83 years), and 40 (22%) of the patients were female.

In the first environmental sampling period, between June to July 2019, 496 environmental samples were obtained across seven time points (25 to 80 sites per time point), from which 20 isolates were determined to be P. aeruginosa. Of these, one tested positive, and one was indeterminate on modified Carbapenem Inactivation Method (mCIM) (both samples were from a shower drain trap). None of these isolates was found to be *bla*_NDM_ positive by Xpert Carba-R.

Subsequently, in the second environmental sampling period (between September and November 2019), 636 environmental samples were obtained across eight time points (77 to 80 sites per time point), from which 155 isolates were found to be P. aeruginosa. Of these, 35 isolates tested positive on mCIM (samples were from shower drain traps [*n* = 14], sinks in a bathroom [steel trap up and below, *n* = 11; P-trap water, *n* = 8], and P-trap water from sinks in patient room [*n* = 2]). Of these, 16 isolates were determined to be *bla*_NDM_ positive by Xpert Carba-R (11 isolates from ward C1 and 5 isolates from ward C2, with both wards located on the same level) and were isolated from samples obtained from three surfaces that include shower drain traps (*n* = 8) and sinks in a bathroom (steel trap up and below, *n* = 4; P-trap water, *n* = 4).

### Genome assembly and MLST profile of *P. aeruginosa* isolates.

We performed *de novo* genome assembly of 199 isolates from hospital C, which comprised a mean assembly size of 6,960,246 bp (6.9 Mb), a mean GC content of 65.99%, and an average of 6,332 protein-coding sequences. Of the 199 P. aeruginosa isolates, 195 (97.9%; 179 clinical and 16 environmental) were assigned to ST308, two belonged to ST1654 and ST2613, while the remaining two isolates were novel STs. All but one of the 195 P. aeruginosa ST308 isolates were *bla*_NDM-1_ positive. For completeness, this *bla*_NDM-1_-negative isolate was included in phylogenetic and resistome analyses. In addition, *de novo* genome assembly was performed on 71 *bla*_NDM-1_-positive ST308 isolates from two other local hospitals (hospitals A and B) in Singapore and 85 *bla*_NDM-1_-negative ST308 global isolates for downstream analysis (see Tables S1 and S2).

### Phylogenetic analysis of ST308 isolates.

**FIG 1:**
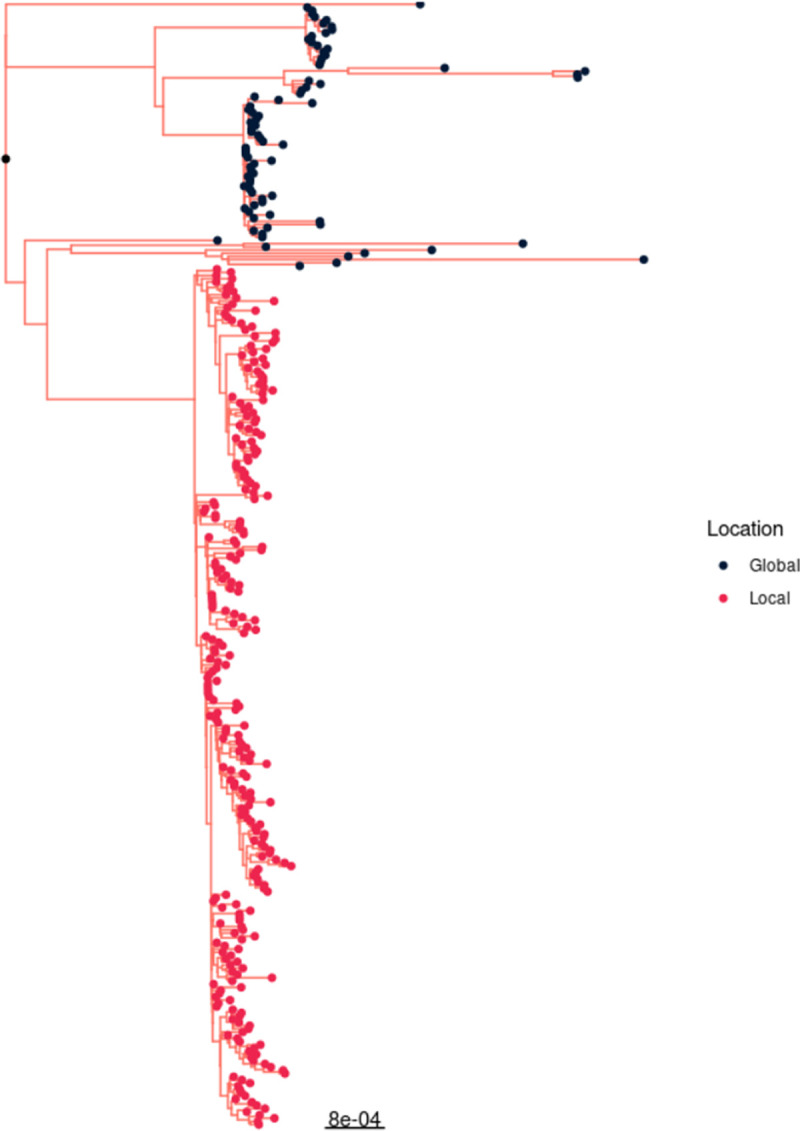
Maximum-likelihood phylogenetic tree with local and global P. aeruginosa ST308 genomes.

We constructed a maximum-likelihood phylogenetic tree to elucidate the phylogenetic relationships between the 266 local *bla*_NDM-1_-positive (31 from hospital A, 40 from hospital B, and 195 from hospital C) and 83 global *bla*_NDM-1_-negative ST308 genomes originating from Germany, France, and Spain. The results revealed that all local *bla*_NDM-1_-positive ST308 isolates from Singapore form a unique and distinct clade from that of global isolates (Fig. 1).

### Emergence of local *bla*_NDM-1_-positive ST308 clone and transmission analysis.

**FIG 2:**
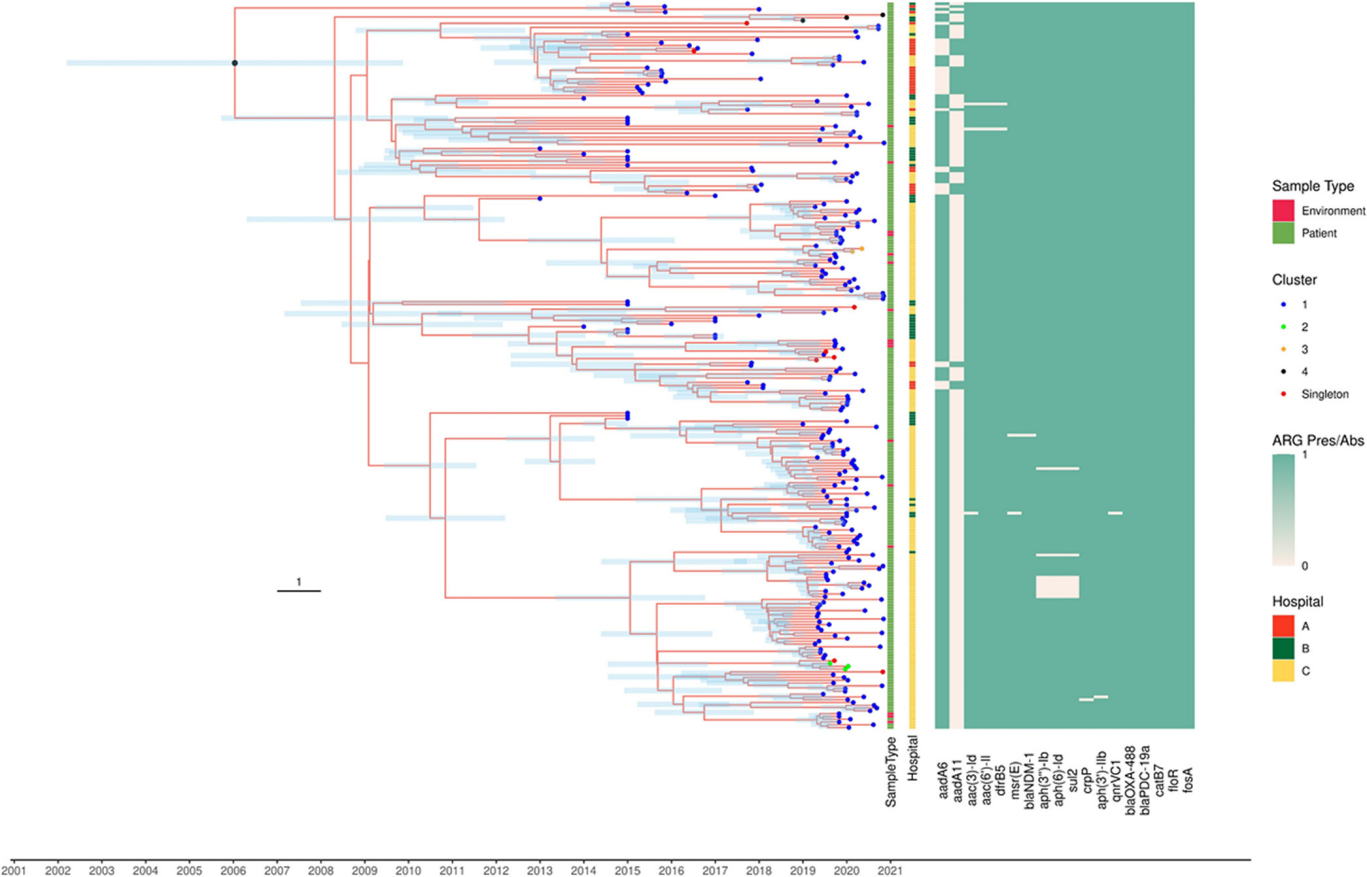
Bayesian maximum clade credibility tree with 261 patient and environmental isolates from local hospitals A, B, and C. Horizontal bars (light blue) on nodes represent posterior probability values and 95% credibility intervals. The color of the tree tip indicates the cluster number the isolate belongs to. Information on the corresponding sample type, hospital, and resistant gene presence/absence matrix are indicated on the right of the tree tip. The presence or absence of ARGs is represented as a heatmap, with green indicating the presence of the corresponding genes, respectively. For completeness, the single *bla*_NDM-1_-negative ST308 P. aeruginosa isolate was included in the phylogenetic tree. The scale represents the number of years.

We detected a temporal signal with a positive correlation between root-to-tip distance versus sample time for the 261 local ST308 isolates in the alignment, indicating suitability for molecular clock analysis to estimate divergence time and substitution rate. Five isolates were excluded from divergent time analysis since sampling dates were not available. The analysis estimated that the initial divergence of local *bla*_NDM-1_-positive ST308 clone in Singapore occurred in the year 2005 (95 % highest posterior density [HPD]: 2001 to 2008) (Fig. 2), which was approximately 8 years earlier than the oldest isolate from the present collection (hospital B in 2013). The substitution rate of *bla*_NDM-1_-positive ST308 clone’s genome was estimated to be 2.421 × 10^−7^ (95% HPD interval, 2.0522 × 10^−7^ to 2.7958 × 10^−7^) substitutions/site/year, equivalent to 1.75 single nucleotide polymorphisms (SNPs)/genome/year.

Four clonal transmission clusters were identified, of which the largest cluster with 245 samples involved all three hospitals (A, B, and C). One cluster (three isolates) involved two hospitals (B and C), while two clusters (encompassing two and three isolates each) were limited to hospital C. Eight isolates (two isolates from hospital B and six isolates from hospital C) did not belong to any cluster (Fig. 2).

### Comparison of integration events in the local and global ST308 genomes.

**FIG 3:**
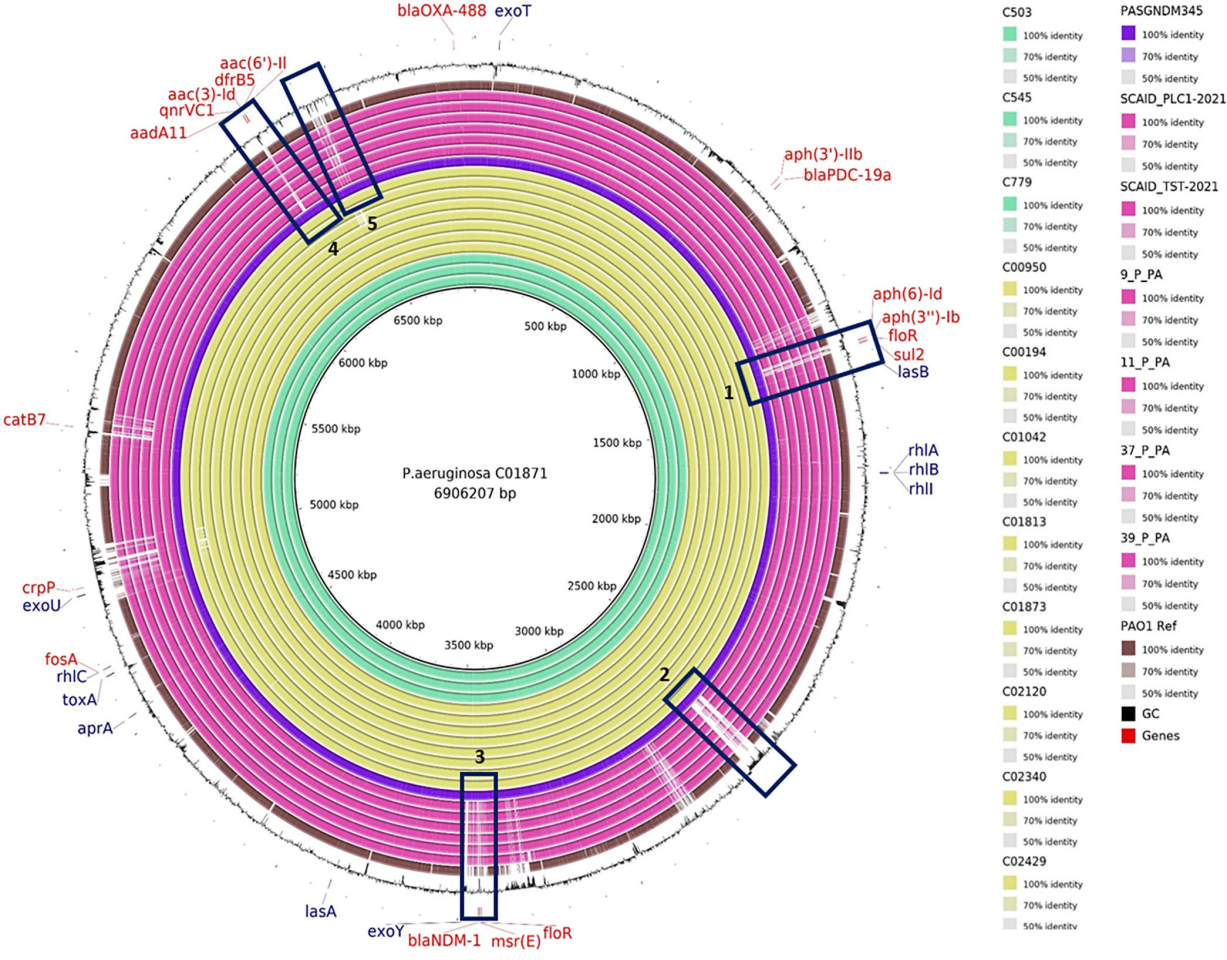
Linear comparison of genomic assemblies of P. aeruginosa involving local *bla*_NDM-1_-positive ST308 environmental isolates (cyan) and clinical isolates (yellow-green) from hospital C with global *bla*_NDM-1_-negative ST308 isolates (pink) and PAO1 reference genome (brown). ARGs (labeled in red) and virulence genes (labeled in purple) are indicated at their respective genomic locations. Cassettes 1, 3, and 4 represent ARG regions, cassette 2 indicates WYL domain containing protein region, and cassette 5 represents Dobby phage integration in local ST308 genomes. To demonstrate the missing cassettes in global samples with higher confidence, two additional global *bla*_NDM-1_-negative ST308 isolates (SCAID PLC1-2021 and SCAID TST-2021) that were not part of the phylogenetic tree analysis have also been included in the visualization.

By comparing all 266 local ST308 isolates with 85 global isolates, including two additional global *bla*_NDM-1_-negative ST308 isolates (SCAID PLC1-2021 and SCAID TST-2021), at least five genomic regions were uniquely present in the local ST308 (Fig. 3). Three integration events incorporated ARGs such as the *floR* gene (cassette 1), *bla*_NDM-1_ with the *msr*(*E*) and *floR* genes (cassette 3), and the *qnrVC1* gene (cassette 5). The two non-ARG genomic regions included WYL domain-containing protein region (cassette 2) and Dobby phage (cassette 5), which were also detected in a minor number of global ST308 isolates. Dobby phage was present in two global isolates with minimum coverage of 99.4% (SNP range, 210 to 212) and WYL domain-containing protein region in eight global isolates with a minimum coverage of 90.3% (SNP range, 1 to 3).

**FIG 4:**
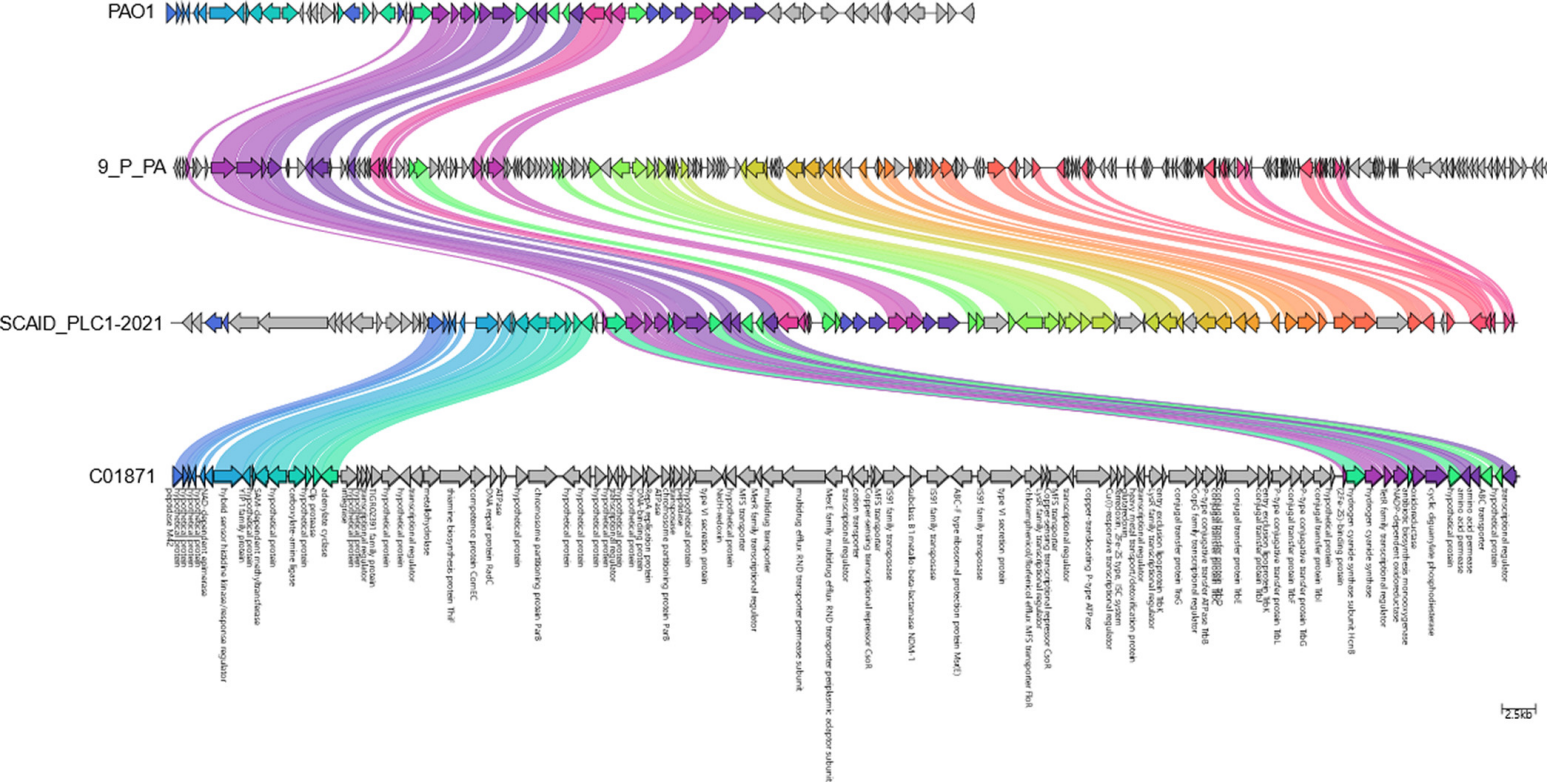
Linear comparison of *bla*_NDM-1_ gene region in local ST308 isolate with global ST308 isolates.

The local ST308 isolates carried *bla*_NDM-1_ with *msr*(*E*) and *floR* within the same gene cassette, which was integrated chromosomally (Fig. 4). Mapping the short reads of the 266 isolates against ICETn_4371_6385 region (73.4 kb), a previously identified integrative and conjugative element (ICE) in carbapenem-resistant P. aeruginosa (PASGNDM345), revealed that 99.2% of the isolates (264/266) harbored the ICETn_4371_6385 region with a minimum of 98.5% coverage and an SNP range of 1 to 112 SNPs (see Table S3). While isolate C01094 carried all three genes, the gene cassette only had 68% coverage of ICETn_4371_6385 region. Isolate C00337 did not carry both *msr*(*E*) and *bla*_NDM-1_, despite possessing 85% coverage of the ICETn_4371_6385 region.

A second copy of *floR* gene (referred to here as *floR_t2*) encoding florfenicol resistance was found incorporated ~2.06 Mb away from the ICETn_4371_6385 among local ST308 isolates within the closed genomes. The *floR* gene showed 99% coverage and 80% identity with the *floR_t2* gene based on pairwise NCBI BLAST alignment results. The *floR_t2* genomic region (26.4 kb) also carried other genes encoding *sul2*, *aph(3″)-Ib*, and *aph(6)-Id* and mercury resistance encoding genes (see Fig. S1). Mapping of the short reads against *floR_t2* region revealed that 96.2% of the isolates (256/266) harbored *floR_t2* region with a minimum of 91.1% coverage and an SNP range of 1 to 115 (see Table S4). In addition, a quinolone-resistant *qnrVC1* gene region (12.2 kb) composed of class 1 integron integrase followed by the aminoglycoside *aac(3)-I* family gene and universal stress protein, *qnrVC1*, as well as a set of hypothetical proteins and transposase genes (see Fig. S2). Around 99.2% of the ST308 isolates (264/266) harbored *qnrVC1* region with a minimum of 92.9% coverage and an SNP range of 1 to 2 (see Table S5). Comparison of C01871 *qnrVC1* and its flanking regions to Brazil V. cholerae strain VC627 gene region (accession number MH782277) revealed that the regions were 99% identical.

Whole-genome alignment also revealed two other genomic regions that were integrated in the chromosome: Dobby, a ϕCTX-like P. aeruginosa-infecting phage isolated from kidney stones, and WYL domain-containing protein (9). Dobby was incorporated in majority of the local ST308 P. aeruginosa isolates (212/270). Short-read mapping of ST308 isolates from three local hospitals against Dobby revealed the presence of the phage in 100% of isolates (31/31) from hospital A, 95% of isolates (38/40) in hospital B, and 71.8% of isolates (143/199) in hospital C, with at least 95.8% coverage of the Dobby ϕCTX-like phage genome and an SNP range of 1 to 142 SNPs (see Table S6 and Fig. S3). The second genomic region of size 55 kb carrying WYL domain-containing protein possessed 39 genes, of which 23 encode hypothetical proteins and two integrase and three transcriptional regulators, including the *alpA* family transcriptional regulator. In addition, genes encoding 7-cyano-7-deazaguanine synthase, alkaline phosphatase, autotransporter domain-containing protein, DNA replication, and repair protein RecF, as well as globin, helicase, hydrolase, or metal-binding protein, hydrolase TatD, restriction endonuclease subunit M, and serine/threonine protein kinase, were also identified (see Fig. S4). Mapping the short reads revealed that all of the 266 local ST308 isolates harbored WYL domain-containing protein region with a minimum of 96.1% coverage and an SNP range of 1 to 2 (see Table S7). Taken together, the result suggests that this region is involved in cell maintenance by encompassing genes involved in metabolic pathways, gene expression, and transport.

### Core genome, resistome, and virulome analysis of all *bla*_NDM-1_-positive ST308 clone.

The core genome size of P. aeruginosa was 6.03 Mb with 5,952 genes common in at least 95% of isolates representing the core gene set for all the 261 local isolates (see Fig. S5).

A total of 18 ARGs were detected in all 266 local ST308 isolates, including ARGs conferring resistance to aminoglycosides [*aac(3)-Id*, *aac(6′)-Il*, and *aph(3′)-Iib*], beta-lactams (*bla*_OXA-488_), carbapenems (*bla*_NDM-1_), cephalosporins (*bla*_PDC-19a_), chloramphenicol and its derivative florfenicol (*catB7*, *floR*), fluoroquinolones (*crpP*), fosfomycin (*fosA*), macrolides [*msr*(*E*)], quinolones (*qnrVC1*), streptomycin [*aph(6)-Id*, *aph(3″)-Ib*, *aadA6*, and *aadA11*], sulfonamides (*sul2*), and trimethoprim (*dfrB5*) (see Fig. S6). Of the 18 ARGs, 16 ARGs were conserved in at least ~95% of the ST308 local isolates, while the remaining two ARGs, *aadA6* and *aadA11*, were found in ~88% (*n* = 235) and ~12% (*n* = 31) of the isolates, respectively. Alignment of genome assemblies against the reference PASGNDM345 closed circular genome confirmed that all the ARGs were located in the bacterial chromosome. The *aadA6* gene was detected in isolates from hospitals B and C but was found to alternate with another aminoglycoside adenylyltransferase, *aadA11*, in hospital A. Among the local ST308 isolates, resistant gene *floR* was present in 95.4% (*n* = 254) of isolates as two chromosomal gene copies. ARGs in the predominant ST308 clone, as well as genomes from other STs, are listed in Table S8.

Overall, a wide variety of genes related to virulence were found among the studied P. aeruginosa isolates. Of the 238 virulence genes found in ST308 clone, 221 were conserved in at least ~95% of the isolates (see Table S9). Analysis showed that ST308 clones harbored alkaline metalloproteinase (*aprA*; 99.6% samples), elastase (*lasB*; 99.6% samples), exotoxin A (*toxA*; 100% samples), exoenzyme U (*exoU*; 99.6% samples), and GDP-mannose 6-dehydrogenase (*algD*; 100% samples). Virulence gene *exoS* was only detected in one isolate.

### Environmental isolates interspersed.

Environmental sampling of four wards in Hospital C identified 16 *bla*_NDM-1_-positive P. aeruginosa isolates. The isolates were sampled from the shower drain traps (*n* = 8), and sinks in the bathroom (steel trap up and below, *n* = 4; P-trap water, *n* = 4). Together with clinical and surveillance sequences from all three hospitals, phylogenetic analysis revealed that the environmental isolates were interspersed and do not form a distinct clade (Fig. 2).

## DISCUSSION

High-risk P. aeruginosa ST308 associated with the production of multiple carbapenemases, mainly *bla*_IMP_ and *bla*_VIM_, has been shown to exhibit global dissemination (4, 10). However, circulation of a *bla*_NDM-1_-positive ST308 clone has only been reported in Singapore and Malaysia (5, 6, 8). Our study provides new insights into the genomics and transmission of *bla*_NDM-1_-positive P. aeruginosa among Singapore hospitals.

We examined the phylogenetic relationship of locally circulating *bla*_NDM-1_-positive ST308 isolates with global *bla*_NDM-1_-negative ST308 isolates originating from Germany, France, and Spain to assess the uniqueness of the local ST308 clone. Phylogenetic analysis revealed two distinct clades for global and local ST308 genomic sequences, while molecular clock analysis estimated that the *bla*_NDM-1_-positive ST308 clone was introduced in Singapore in the early to middle 2000s, with limited epidemiological data documenting the presence of this clone in the neighboring country of Malaysia, where a single isolate was documented. The estimated substitution rate of *bla*_NDM-1_-positive ST308 clone was similar to the evolutionary rate of P. aeruginosa ST308 isolates reported previously (11). Majority of isolates from three different tertiary care hospitals formed a single large clonal transmission cluster, strongly supporting cross transmission between the three health care institutions.

The *bla*_NDM-1_-positive ST308 clone in Singapore differed from other global ST308 sequences in that several gene cassettes containing ARGs were chromosomally integrated. ARGs were mainly found in three gene cassettes with *aph(6)-Id*, *aph(3″)-Ib*, *floR*, and *sul2* genes in cassette 1, *bla*_NDM-1_, *msr*(*E*), and *floR* genes in cassette 3 (ICETn_4371_6385), and *aac(3)-Id*, *aac(6′)-Il*, *aadA6/aadA11*, *dfrB5*, and *qnrVC1* genes in cassette 4. The presence of *bla*_NDM-1_ in ICETn_4371_6385 in P. aeruginosa was previously reported in another local study (12). Unlike *Enterobacterales*, where plasmid-mediated transmission is a major mode of ARG dissemination, prior research suggests that chromosomal integration of ARGs plays a larger role in ARG dissemination in P. aeruginosa (13).

The *bla*_NDM-1_-positive P. aeruginosa ST308 clone carried a wide variety of virulence factors, which could potentially contribute to its ability to cause disease and invade sterile sites. ExoU and ExoS, effector proteins of the type III secretion system that contributes to cellular cytotoxicity and invasion, were among the virulence genes detected (14). Other virulence factors such as elastase *lasB* (known to evade the immune response and target alveolar macrophages), T3SS and T6SS (facilitator of colonization), rhamnolipids, phenazines, cyanide, exotoxin A, elastase (genes involved in promoting the impact of bacterial cytotoxicity on host tissue), and *algD* (involved in bacterial adhesion) were also present.

Phylogenetic analysis of patient and environmental samples suggest transmission between humans and environment. The high genome similarity in gene content between ST308 clinical and environmental isolates accompanied with a very low pairwise SNP difference among the isolates supports possible cotransmission of *bla*_NDM-1_-positive ST308 clones between the two sources. Several studies have described outbreaks of hospital-acquired P. aeruginosa infections with localization of the bacteria detected on health care devices, as well as various environmental sites, supporting a role of the hospital environment as a reservoir in the transmission of carbapenemase-producing P. aeruginosa (15, 16).

This study has several limitations. We did not have access to data on patient characteristics (including comorbidities), inpatient diagnosis, and treatment histories to facilitate virulence assessment. However, the presence of P. aeruginosa isolated from sterile sites (e.g., the bloodstream) demonstrates its virulence potential. We also did not have data on the movement of patients to understand the transmission dynamics. Samples collected from the three hospitals were not contemporaneous, and future studies for multiple institutions over the same time-period would allow us to better examine spread of *bla*_NDM-1_-positive P. aeruginosa ST308 in health care settings and across the population.

In conclusion, MDR clones such as P. aeruginosa ST308 harboring *bla*_NDM-1_ are a serious nosocomial threat in the health care setting. Our findings document a pathogenic *bla*_NDM-1_-positive P. aeruginosa ST308 clone which has disseminated across the three sampled institutions with possible environmental reservoirs contributing to transmission. Surveillance in Singapore and beyond for dissemination is essential to determine whether existing measures are sufficient to control spread of this ST308 clone.

## MATERIALS AND METHODS

### Isolate selection.

From April 2019 and November 2020, a total of 1,567 rectal/stool swabs taken from patients in hospital C were screened directly for five common carbapenemase genes (KPC, NDM, VIM, IMP-1, and OXA-48) via the Xpert Carba-R (Cepheid, USA), of which 88 swabs were *bla*_NDM_ positive. In addition, 95 *bla*_NDM_-positive P. aeruginosa from various clinical specimens were identified through clinical cultures, and the presence of *bla*_NDM_ was confirmed via Xpert Carba-R analysis performed on the isolate.

Environmental sampling was conducted over two time periods. The first sampling period was between June and July 2019, and the second sampling period was between September and November 2019. Environmental samples were collected from up to 10 sites per room in 72 rooms across four different wards (C1, C2, C3, and C4). These surfaces included sinks in patient rooms (external surface and internal bowl, steel trap up and below, and P-trap water), sinks in bathrooms (external surface and internal bowl, steel trap up and below, and P-trap water), sinks in anterooms (external surface and internal bowl, steel trap up and below, and P-trap water), and shower drain traps. Sampling was conducted over two different time periods. During both the first (between June and July 2019) and second (between September and November 2019) time periods, a weekly sampling was carried out, resulting in seven and eight time points, respectively. Samples were subjected to meropenem-disk tryptic soy broth enrichment, followed by culture on MacConkey agar (17). mCIM was performed for detection of carbapenemase-producing organisms, and then the presence of carbapenemase genes was identified by using Xpert Carba-R.

*bla*_NDM_-positive isolates as detected above were cultured on tryptic soy agar/sheep blood/MacConkey agar for 16 to 18 h, and genomic DNA was extracted by using a MagNA pure compact nucleic acid isolation kit I or a Qiagen QIAamp DNA minikit (both according to the manufacturers’ instructions).

### Whole-genome sequencing and genome assembly.

Whole-genome sequencing (WGS) was performed using the Illumina-NovaSeq 6000 platform with 2 × 150-bp paired-end reads for the 199 isolates from hospital C. Short-read sequence data of 71 local *bla*_NDM-1_-positive ST308 isolates originating from hospital As and B, as well as 85 global *bla*_NDM-1_-negative ST308 isolates, were downloaded from publicly available sequence databases (the NCBI Sequence Read Archive [SRA] and European Nucleotide Archive [ENA]) (5, 6, 18–22). Of these 85 isolates, 79 isolates were sequenced using Illumina, 4 were sequenced using both Illumina and Nanopore, and 2 were sequenced using Pacific Biosciences sequencing platforms. Unless otherwise specified all the analysis tools were ran with default parameters. Quality control for Illumina reads was performed using FastQC (v0.11.7), followed by adapter trimming and quality filtering using BBDuk (v38.11) with the parameters “ktrim = r k = 23 mink = 11 hdist = 1 qtrim = rl trimq = 30 minavgquality = 30” (23, 24). *De novo* assembly of the short-read sequences was performed using SPAdes (v3.13.0; parameter: –careful) and contigs of <1,000 bp were excluded from downstream analysis (25). A representative set of 12 isolates from different clades were selected for sequencing on GridION (Oxford Nanopore Technologies) using a rapid barcoding kit (SQK-RBK004). Demultiplexing and adapter trimming of the long-read sequence data were performed using qcat (v1.1.0; https://github.com/nanoporetech/qcat). Hybrid assemblies were performed using Unicycler (v0.4.9) and polished using short-reads with Pilon (26, 27), and the long-read assemblies were performed using Raven (v1.5.0) (28).

### Genome analyses.

Multilocus sequence typing (MLST; v2.19.0) was used for species assignment and determination of ST profiles (29). Kraken2 (v2.0.8-beta) was utilized for isolates that could not be characterized by MLST, while isolates with no ST assignment were processed using the CGE Bacterial Analysis Pipeline (https://cge.cbs.dtu.dk/services/cge/) (30). All assembled genome sequences were screened for antibiotic resistance genes by a standalone local version of ABRicate (v1.0.1; database: “ncbi”) (31). SRST2 (v0.2.0) was used to identify presence of integration events in ST308 clone (32). SRST2 with the “–min_coverage” parameter was set to 60 to identify coverage of integration events and to confirm the absence of the *bla*_NDM-1_ gene.

Genome annotation was performed with Prokka (v1.14.6) and the pangenome was reconstructed using Roary (v1.007002; with the parameters -e –mafft -g 60000 -p 48 -cd 95) (33, 34). A consensus reference sequence was generated using the core genome alignment of the local ST308 isolates from Roary, which was used as an input reference for mapping the short reads using Snippy (v4.4.3) (35). Subsequently, the alignment from snippy was used as an input to Gubbins (v2.3.4) to filter recombination (36). The resulting polymorphic SNP sites were used to calculate the SNP differences between the isolate pairs.

### Phylogenetic analysis and divergence time estimation.

SNP alignment generated by Gubbins was used as an input to IQ-TREE2 (v2.1.3) to reconstruct a maximum-likelihood tree (37). The substitution rate and the most recent common ancestor was then estimated using BEAST (v2.5) with a strict molecular clock model and bModelTest as the nucleotide substitution model (38, 39). *bla*_NDM-1_-positive ST308 isolates originating from local hospitals with culture date available were included for the divergence time estimation analysis. Three independent BEAST runs of 100 million cycles resulted with all the parameters possessing ESS values >200 confirming the run convergence. The resultant log files from the three independent runs were combined using LogCombiner (v2.5.2) program, and a maximum clade credibility tree was reconstructed by combining trees using the tree annotator program (39). Individual genome assemblies were compared using BRIG (v0.95), and genomic regions were visualized using clinker (v0.0.23) (40, 41). PASGNDM345, the first local *bla*_NDM-1_-positive ST308 P. aeruginosa genome detected and characterized in Singapore, which also harbored the integrative and conjugative element (ICE) ICETn_4371_6385, was used for genomic annotation (12). A phylogenetic tree and corresponding heatmap was visualized using R package ggtree (v3.3.0) (42).

### Transmission analysis.

Clonal transmission among ST308 clone was established between the isolates as long as they carried the same carbapenemase gene allele and had pairwise SNP count (based on the recombination-filtered core gene alignments) below the BEAST-derived mutation rate threshold, assuming a Poisson distribution for the accumulation of mutations (43). Isolates were considered members of a clonal transmission cluster if the isolate met criteria for clonal transmission with at least one other member in the clonal transmission cluster.

### Ethical approval.

This study was reviewed and approved by ethics institutional review boards of National Health Group Singapore (DSRB reference 2019/01071), which did not require that patients provide written informed consent.

### Data availability.

All raw sequencing reads were deposited in the NCBI Sequence Read Archive (SRA) database under BioProject accession number PRJNA811722. ST308 sequence data for hospitals A and B were downloaded from NCBI SRA under BioProject accession numbers PRJNA507901 and PRJNA656645. Sequence data for global ST308 isolates downloaded from NCBI SRA and European Nucleotide Archive (ENA) are listed in Table S2 in the supplemental material.
