# Supplementary material for: Dissemination of Pseudomonas aeruginosa blaNDM-1-Positive ST308 Clone in Singapore
Source: Microbiol Spectr. 2023 Apr 12;11(3):e04033-22. doi: 10.1128/spectrum.04033-22 (PMC10269627; doi:10.1128/spectrum.04033-22)
Supplement: Supplemental file 1 — Fig. S1 to S6. Download spectrum.04033-22-s0002.pdf, PDF file, 2.1 MB [file spectrum.04033-22-s0002.pdf]

Dissemination of *Pseudomonas aeruginosa* *bla*$_{NDM-1}$-positive ST308 clone in Singapore

Supplementary Figures

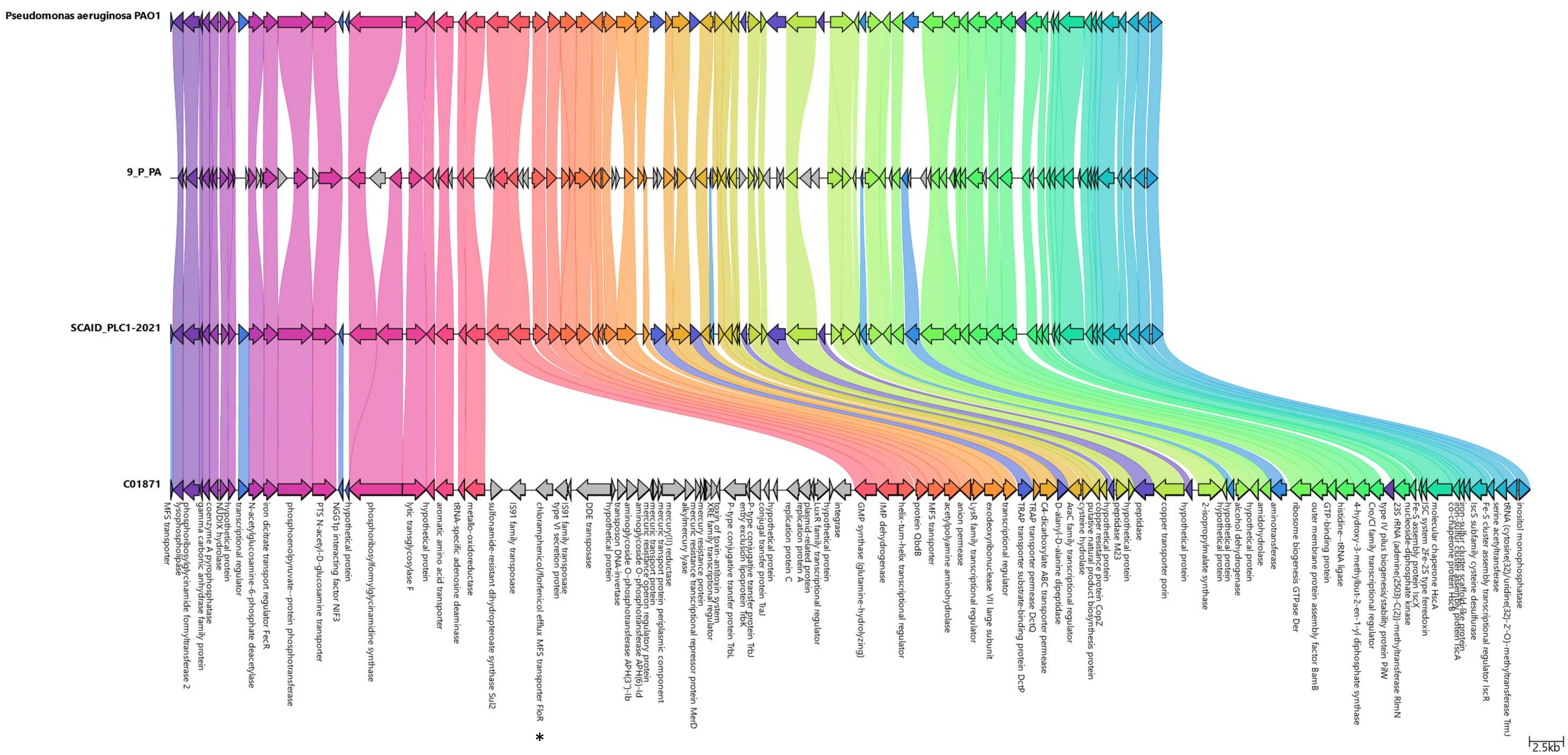

Figure S1. Linear maps of *floR_t2* gene region in local ST308 isolate (C01871) and global ST308 isolates. Genes are shown in arrows with distinct colors. The length of each arrow is to the scale of the gene size, whereas the arrow direction indicates the transcriptional direction of the gene. The *florR* gene of interest is indicated with an asterisk (*).

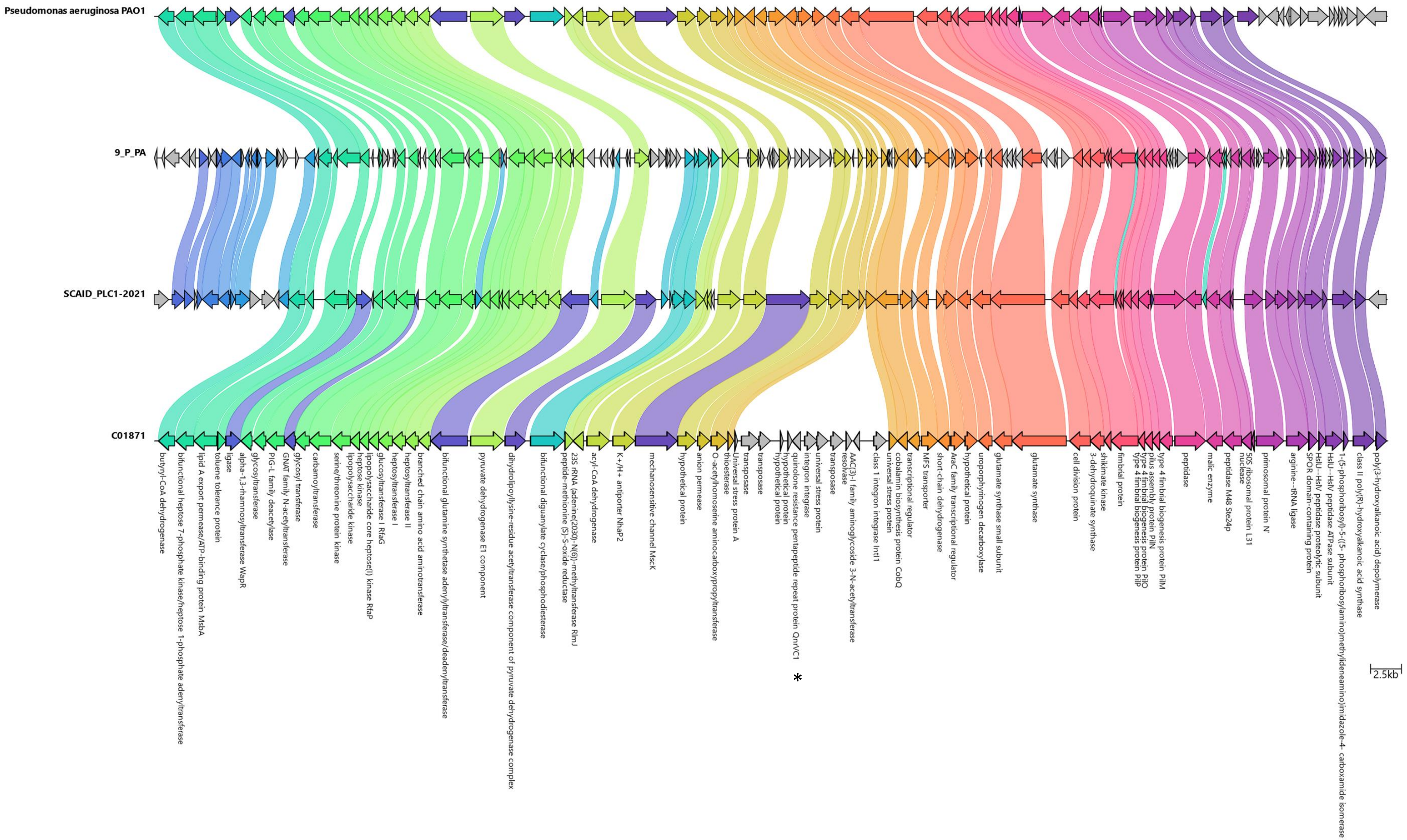

Figure S2. Linear maps of *qnrVC1* gene region in local ST308 isolate (C01871) and global ST308 isolates. Genes are shown in arrows with distinct colors. The length of each arrow is to the scale of the gene size, whereas the arrow direction indicates the transcriptional direction of the gene. The *qnrVC1* gene of interest is indicated with an asterisk (*).

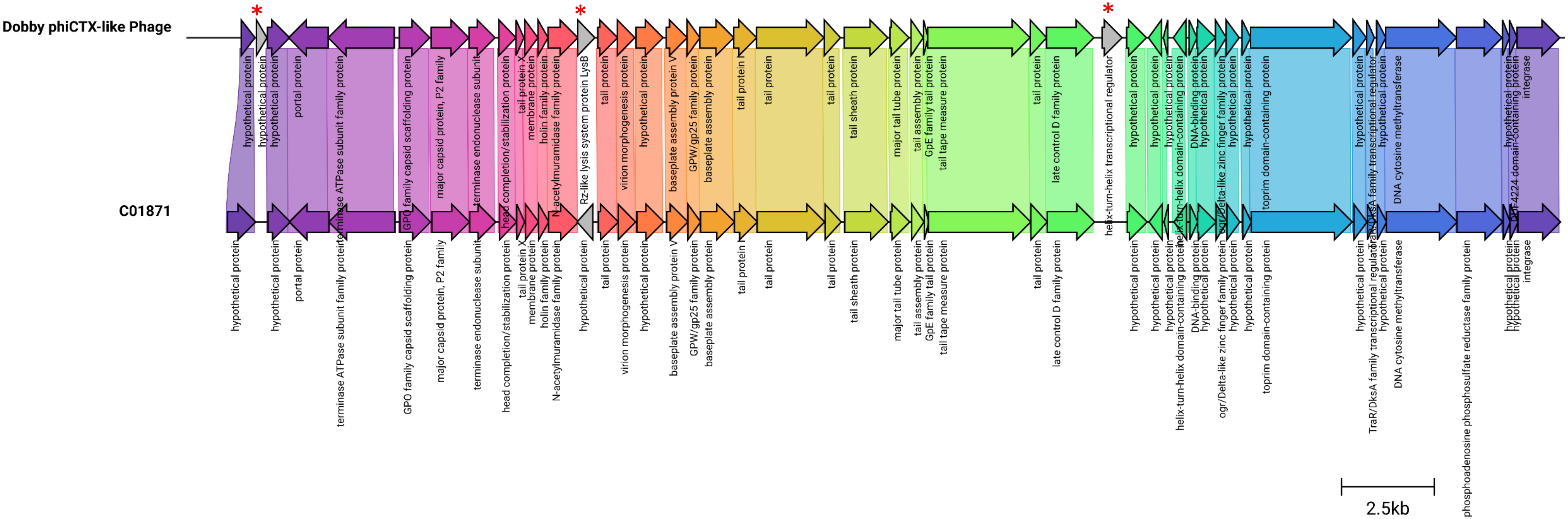

Figure S3. Linear map of Dobby, a φCTX-like *P. aeruginosa*-infecting phage and local ST308 isolate (C01871). Genes are shown in arrows with distinct colors. The length of each arrow is to the scale of the gene size, whereas the arrow direction indicates the transcriptional direction of the gene. The genes indicated with asterisk (*) were confirmed to be present in local ST308 isolate (C01871) sample with 100% coverage using NCBI-BLAST although they were not detected by *ab initio* prediction using Prokka.

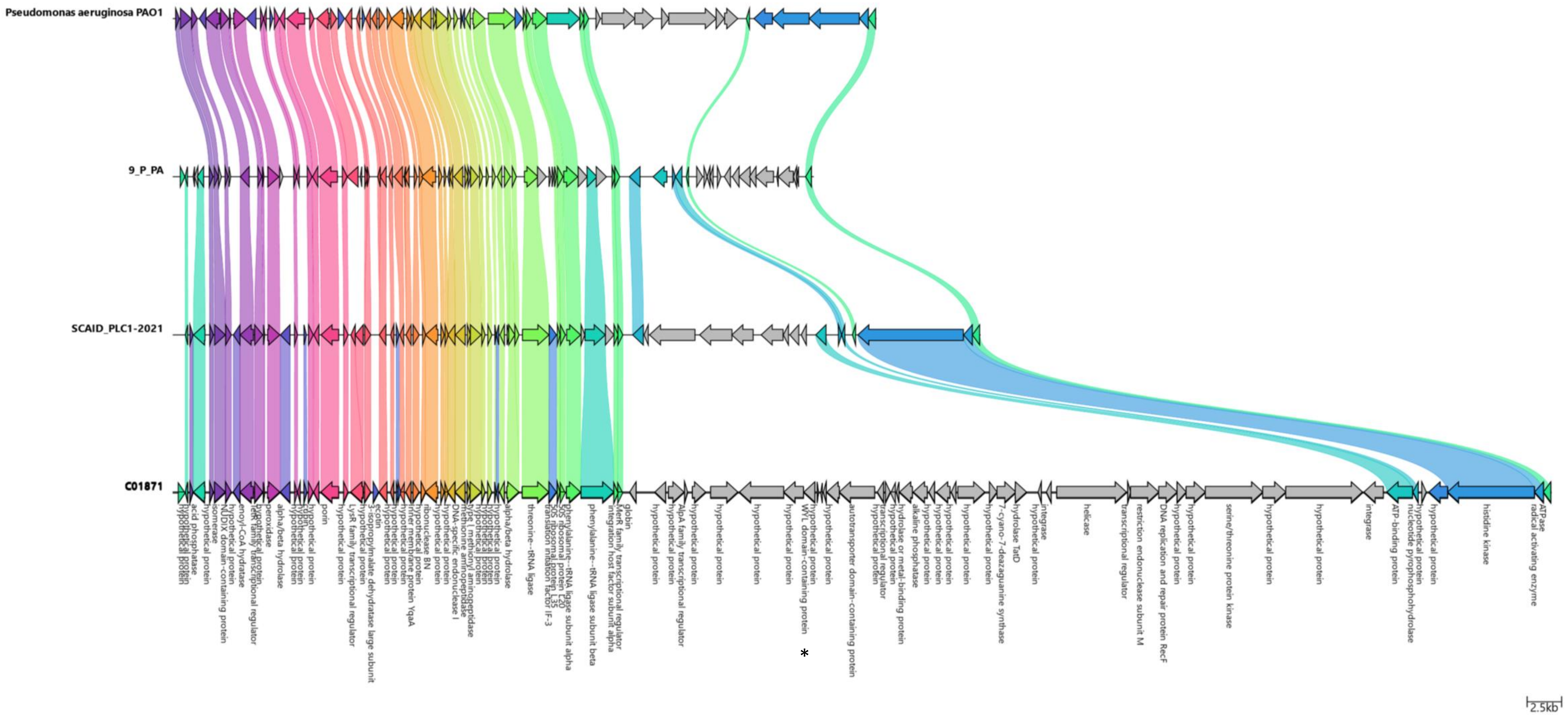

Figure S4. Linear maps of WYL domain-containing protein in local ST308 isolate (C01871) and global ST308 isolates. Genes are shown in arrows with distinct colors. The length of each arrow is to the scale of the gene size, whereas the arrow direction indicates the transcriptional direction of the gene. The WYL domain-containing protein of interest is indicated with an asterisk (*).

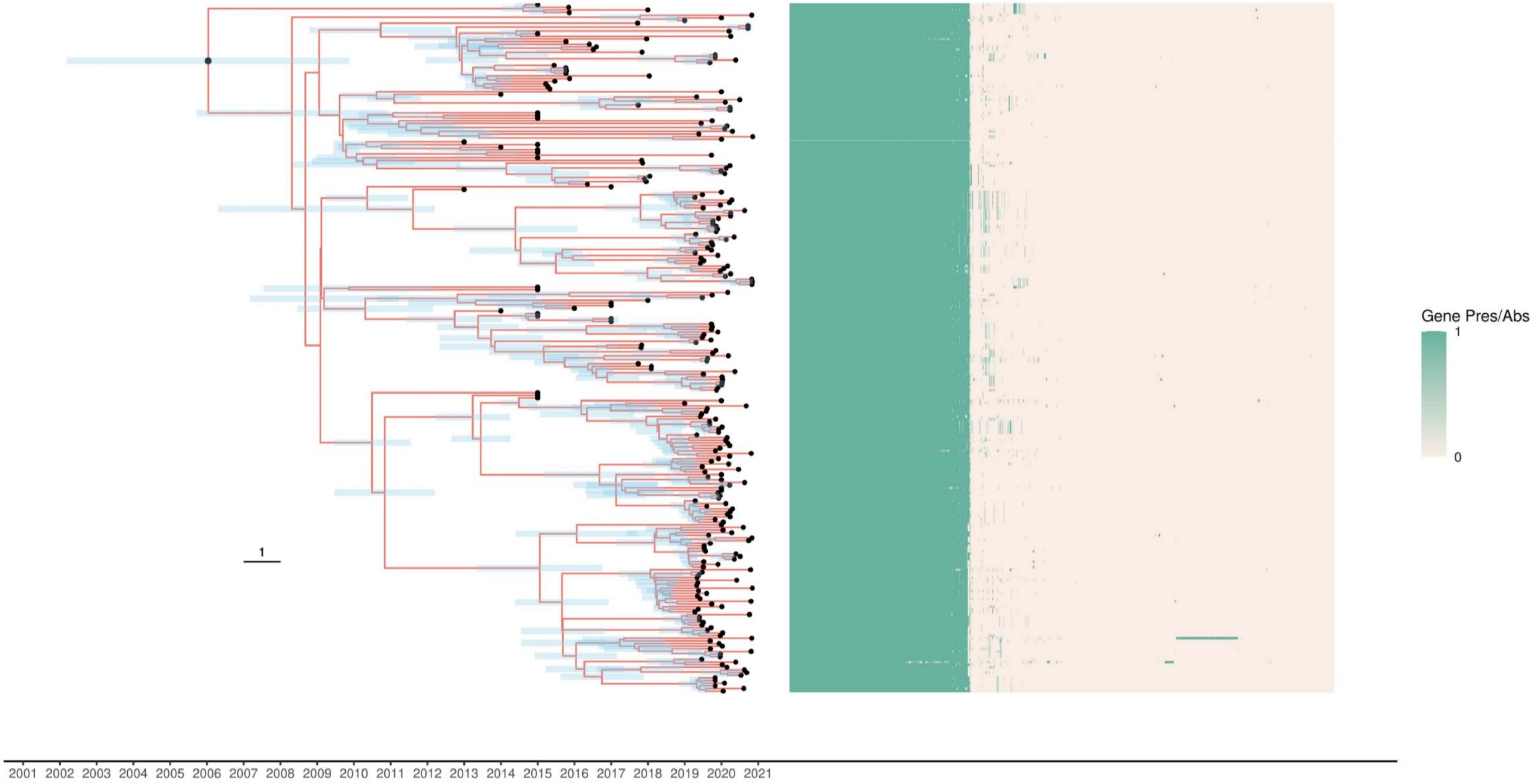

Figure S5. Bayesian maximum clade credibility tree with 261 *bla*$_{NDM-1}$-positive ST308 *P. aeruginosa* patient and environmental isolates from local hospital A, B and C. Horizontal bars (light blue) on nodes represent posterior probability values and 95% credibility intervals. Pan genome matrix is presented in the presence/absence heatmap, with green indicating presence of the corresponding genes. For completeness, the single *bla*$_{NDM-1}$-negative ST308 *P. aeruginosa* isolate was included in the phylogenetic tree. Scale represents number of years.

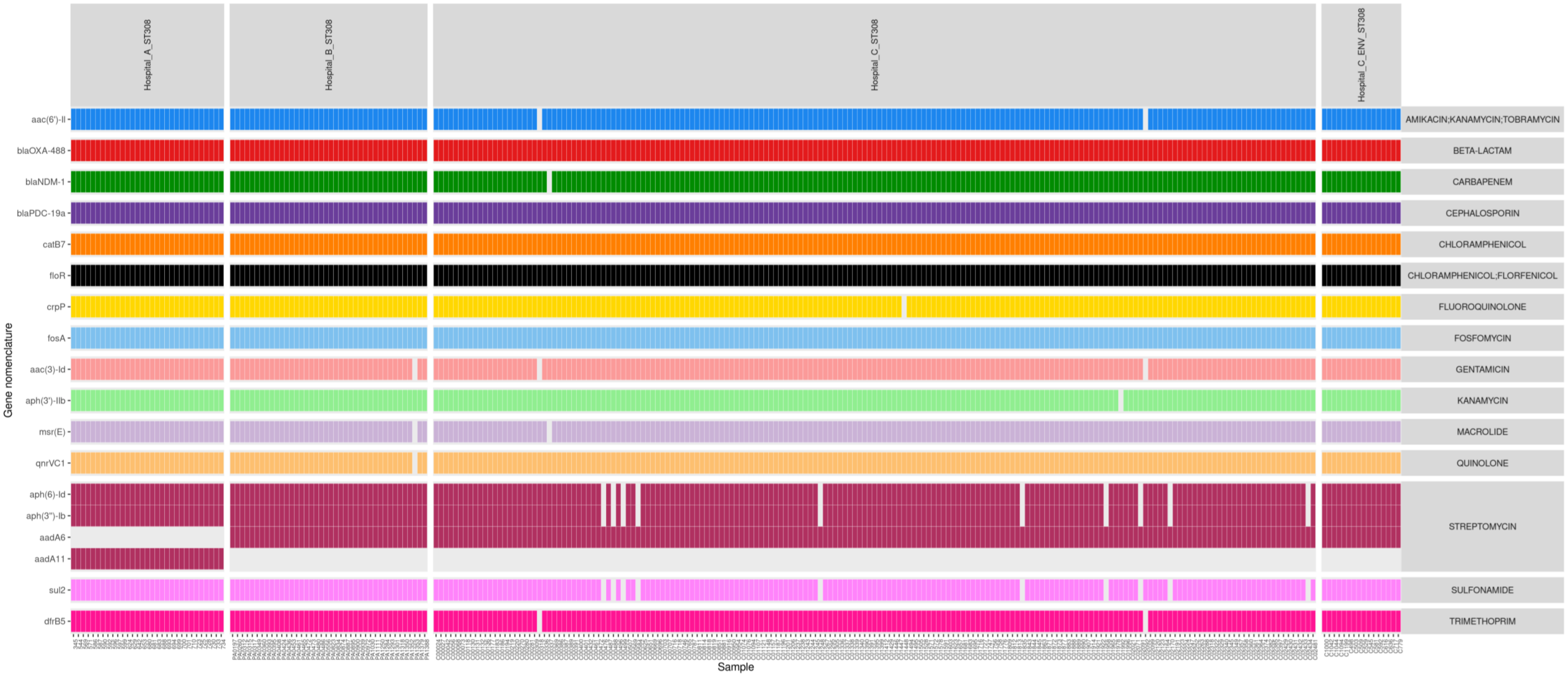

Figure S6. Antimicrobial resistance phenotype of 266 bla_{NDM-1}-positive ST308 *P. aeruginosa* local isolates across the three local hospitals. For completeness, the single bla_{NDM-1}-negative ST308 *P. aeruginosa* isolate was included in the analysis.
